# Cervical Rotatory Manipulation Decreases Uniaxial Tensile Properties of Rabbit Atherosclerotic Internal Carotid Artery

**DOI:** 10.1155/2017/5189356

**Published:** 2017-02-16

**Authors:** Shaoqun Zhang, Ji Qi, Lei Zhang, Chao Chen, Shubhro Mondal, Kaike Ping, Yili Chen, Yikai Li

**Affiliations:** ^1^School of Traditional Chinese Medicine, Southern Medical University, Guangzhou 510515, China; ^2^Affiliated Hospital of Traditional Chinese Medicine, Luzhou Medical University, Luzhou 646000, China; ^3^Zhujiang Hospital of Southern Medical University, Guangzhou 510280, China; ^4^Department of Biostatistics, Southern Medical University, Guangzhou 510515, China

## Abstract

*Objective*. To investigate the effects of one of the Chinese massage therapies, cervical rotatory manipulation (CRM), on uniaxial tensile properties of rabbit atherosclerotic internal carotid artery (ICA).* Methods*. 40 male purebred New Zealand white rabbits were randomly divided into CRM-Model group, Non-CRM-Model group, CRM-Normal group, and Non-CRM-Normal group. After modeling (atherosclerotic model) and intervention (CRM or Non-CRM), uniaxial tensile tests were performed on the ICAs to assess the differences in tensile mechanical properties between the four groups.* Results*. Both CRM and modeling were the main effects affecting physiological elastic modulus (PEM) of ICA. PEM in CRM-Model group was 1.81 times as much as Non-CRM-Model group, while the value in CRM-Model group was 1.34 times as much as CRM-Normal group. Maximum elastic modulus in CRM-Model group was 1.80 times as much as CRM-Normal group. Max strains in CRM-Model group and Non-CRM-Model group were 30.98% and 28.71% lower than CRM-Normal group and Non-CRM-Normal group, respectively. However, whether treated with CRM or not, the uniaxial tensile properties of healthy ICAs were not statistically different.* Conclusion*. CRM may decrease the uniaxial tensile properties of rabbit arteriosclerotic ICA, but with no effect on normal group. The study will aid in the meaningful explanation of the controversy about the harmfulness of CRM and the suitable population of CRM.

## 1. Introduction

Massage therapy (MT), one of the complementary and alternative treatments, is defined as a therapeutic manipulation by trained therapists using the hands or a mechanical device, in which numerous specific and general techniques are often used in sequence, such as effleurage, petrissage, and percussion [1]. MT has a long history, being first described in China around 2700 BC and soon thereafter in India and Egypt [2, 3], and has become popular in the United States and the rest of the world in recent decades [4]. MT may be the earliest and most primitive tool to improve pain. With its popularity in pain relief, it has become a widely accepted treatment for neck, shoulder, and low back pain [3, 5]. However, the increased use of MT also brings attention to its safety. For example, while cervical spine manipulation (CSM), one of the MT, is believed to contribute to rapid recovery from neck pain (NP) [6], several studies have demonstrated an association between CSM and neurovascular injury that can result in stroke or even death [7–9]. Some researchers believe that the frequency of neurovascular injury following spinal manipulative therapy is rare, with a reported occurrence of 1 in 100000 [10] to 1 in several millions [11]. Nevertheless, other researchers hold that the methods for these calculations were often flawed. They found that quite a few cases remain unreported [12] and some serious complications may follow an unrecognized but substantial proportion of CSM therapies [13]. Wand et al. [14] even thought that we should abandon CSM for mechanical neck pain. In 2001, the first case control study looking at the association between stroke and chiropractic manipulation have been published [8]. Furthermore, some similar researches have emerged [7, 9]. In 2007, two large cohort studies, involving chiropractic patients, have been published adding to the evidence that less serious events are the more commonly occurring ones.

However, there are some limitations in previous studies. Firstly, previous studies were mainly to investigate the effects of MT on vascular hemodynamic properties [15, 16] but failed to consider the effect of MT on vascular biomechanic properties. Secondly, the subjects in most of previous studies were healthy and asymptomatic [15] or cadavers [14]; thirdly, most of previous studies were to study the effect of CSM on the vertebral artery [14, 15] but next to nothing on the internal carotid artery (ICA). Most importantly, previous studies mainly studied the safety of the western MT, but few of Chinese MT. Although there are some similarities in MT of different countries, their specific operations are different. We hypothesized that there would be a corresponding change in uniaxial tensile properties of rabbit atherosclerotic ICA after Chinese MT [cervical rotatory manipulation (CRM)], and that may be one of the reasons leading to the occurrence of adverse events.

Restricted by ethics and safety consideration, it is hard to conduct study about this problem in human body. But rabbit model of atherosclerosis has been one of the most common animal models used to replicate atherosclerosis in human body [17]. Taking this into consideration, our research makes intervention of CRM on prepared rabbit arteriosclerotic ICA model, and the purpose of the present study was (1) to explore the effects of CRM and atherosclerotic plaque on uniaxial tensile properties of ICAs in rabbits; (2) to explore the relationship between the internal carotid uniaxial tensile properties of rabbits and the complications following CRM; (3) to explore the suitable population of CRM.

## 2. Materials and Methods

### 2.1. Ethics Statement

All procedures were approved by the Institutional Animal Care and Use Committee of China Academy of Chinese Medicine Science (no. 201506034) and performed in accord with the National Institutes of Health Guide for the Care and Use of Laboratory Animals (Office of Science and Health Reports CPRR/NIH 1996).

### 2.2. Animals

Forty healthy male purebred New Zealand white rabbits aged three months (weight range 2.0–2.4 kg) were housed in a pathogen-free facility in microisolator cages (China Academy of Chinese Medicine Science Experimental Animal Center, Beijing, China). According to random number table method, twenty-four of the forty New Zealand white rabbits were chosen randomly to be the rabbits of model group (*n* = 24), while the remaining were be chosen randomly to be the rabbits of normal group (*n* = 16). After the formation of atherosclerosis model, the model rabbits and the normal rabbits were divided randomly into two groups, respectively. In other words, all the rabbits were randomly divided into four groups: CRM-Model group, Non-CRM-Model group, CRM-Normal group, and Non-CRM-Normal group. The CRM-Model group and CRM-Normal group were treated with CRM in the course of the experiment (5 times, once every three days), while the other two groups were not treated.

### 2.3. Internal Carotid Atherosclerosis Modeling

The ICA atherosclerosis model was established by ICA balloon injury combined with a high-fat diet (1% cholesterol, 7.5% egg yolk, 5% lard, and 86.5% common feed; Beijing Jin Muyang Experimental Animal Feed Science and Technology). The model rabbits were fed with the high-fat diet (120 g/d) and adapted to the environment for 1 week. At the end of the first week, after being anesthetized with sodium pentobarbital (22.5 mg/kg, intravenous injection), the model rabbits underwent balloon injury of the left ICA according to the following method [18]: the 0.67 mm balloon catheter was gently inflated and retracted, and it was pulled repeatedly three times in each model rabbit's ICA. A postoperation muscular injection of ampicillin [50 mg/(kg*∗*d), five days] was given to prevent infection. The high-fat diet was continued for 12 weeks after surgery. During the experiment, the normal rabbits were fed with a regular diet (120 g/day) and the model rabbits were fed with a high-fat diet (120 g/day) until 12th weeks after modeling surgery, and then the model rabbits will be fed with a regular diet (120 g/day) too. All of the rabbits were given free access to water.

### 2.4. Ultrasonography Examination

In the 12th weeks after modeling, left ICA of all rabbits were observed under color Doppler ultrasonography examination. As for preparation, the rabbits were anesthetized by 3% pentobarbital sodium (120 mg/kg, IV) and fixed at dorsal position with shaved skin in neck. Then, by use of color Doppler ultrasonography machine (Philips, IU22) with a mini probe (Philips, L15-7io), left ICAs in rabbits were observed whether atherosclerotic plaques had formed or not. For those ICAs which had atherosclerotic plaques obviously, five positions on these ICAs were chosen randomly to measure the internal carotid atherosclerotic stenosis rate, and the maximum stenosis rate was recorded as the final stenosis rate of this ICA. Most importantly, in order to measure each ICA stenosis rate at the same position after CRM/Non-CRM, the distance between the position of maximum stenosis rate of the ICA and the common carotid artery bifurcation was recorded.

### 2.5. Cervical Rotatory Manipulation

**Figure 1:**
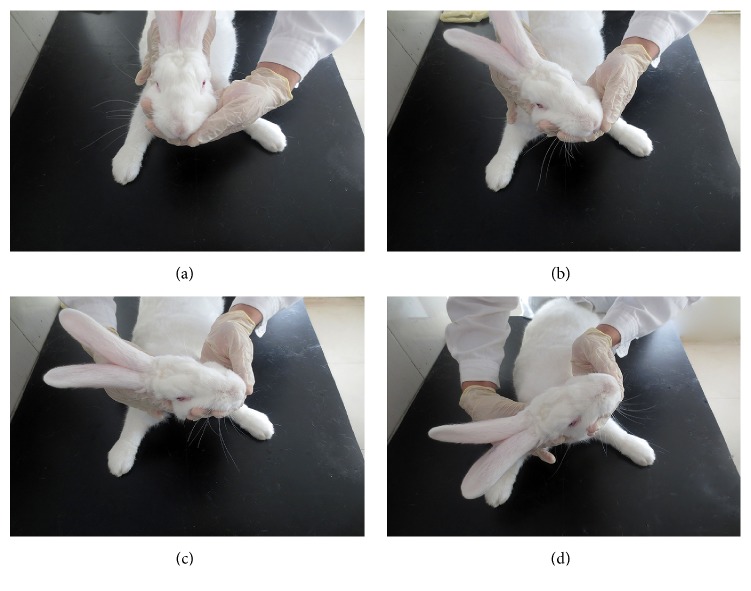
Illustration of the CRM procedure on the rabbit. The process of CRM was from (a)–(d); (a) shows the head and neck of the rabbit in the neutral position; (d) shows the head and neck rotated to the end range of passive motion (about 100~110°).

In this study, one of Feng's spinal manipulative therapies [19], CRM, was selected as the observational spinal manipulative to be studied, which was created by Feng in the 1970s [19] and now is very popular in China. The CRM technique was performed while the rabbit was in a sitting position, and the cervical vertebrae were pressed by a gentle but stabilized torsion force. The CRM technique was similar to any type of traditional spinal manipulation with a focus on loading a torsion force at the involved joints, except more gently and delicately. Only two rotation movements to each side and within the range of motion (ROM) of the cervical spine were applied during a single treatment. During the manipulation, one of the doctor's hands was put under the mandible of the rabbit, while the other hand was put behind the rabbit's occiput. The torsion stress can be quickly loaded on the cervical vertebrae during the rotation, such as in high-velocity and low-amplitude (HVLA) manipulation [20], with the help of doctor's thumb steadily and firmly pushed against the spinous process of the involved segment until the end of the ROM during the process. All of the final torsion angles applied for the CRM were about 100~110° (Figure 1).

### 2.6. Biomechanical Testing

#### 2.6.1. Materials and Specimen Preparation

**Figure 2:**
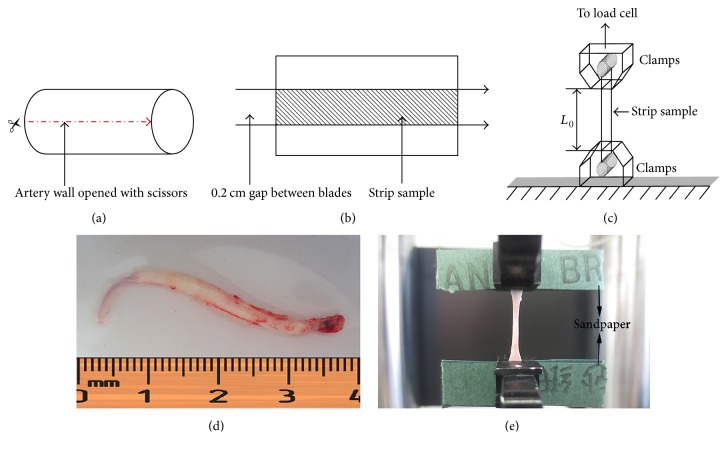
Specimen preparation and unidirectional failure test. (a) and (b) show the division of the segment to produce the strip sample(s). (d) shows the rabbit's left ICA which had formed the atherosclerosis. (c) and (e) show the process of unidirectional failure test. Strip sample of ICA was mounted between two clamps and stretched at a constant rate of elongation. The original length of the strip (*L*_0_) was recorded. The rough sandpapers between the clamps were used to assure a no-slip surface.

At the end of the experiment, all the left ICAs of the rabbits were cut off 2-3 cm for testing. The excessive fat and connective tissue were removed after washing with phosphate-buffered saline (PBS) buffer (in mmol/L): NaCl 150, KCl 2.7, Na_2_HPO 410, NaH_2_PO 42, pH 7.4 [21]. Each artery was then dissected in a similar manner. The artery was opened up longitudinally, and a longitudinal strip, 2 mm wide and 15 mm in length (Figure 2), was then obtained by cutting and immediately was preserved in solution of 0.90% w/v of NaCl at 4°C before the uniaxial tensile test [22].

#### 2.6.2. Experimental Setup and Uniaxial Tensile Test

The tensile test was performed using a unidirectional tensile testing machine (Instron 5848, Micro Tester, Norwood, MA) adapted for testing biological specimens. The strips were taken out from the physiological saline and mounted on the tensile test machine (Figure 2). The initial thickness of the strip (*H*_0_) was measured via a laser beam micrometer (LS-3100, Keyence Corp, Osaka, Japan). Because the thickness of the tissue varied along the strips, five measurements were taken for each strip, and the average value was used as the thickness of each strip. In order to make sure about a firm fixation of samples between the clamps of the machine, a small tensile preload of 0.05 N was applied to each machine, and rough sandpaper was used between the clamps and the sample to assure a no-slip surface [23, 24]. Additionally, in order to minimize the effect of viscoelastic phenomena and obtain constant and reproducible stress-strain curves, each strip was subjected to ten successive cycles before the measurement [25]. The longitudinal force (*F*) applied to the strip and the initial length of the strip (*L*_0_) between the two clamps were measured by the tension device with a frequency of 50 Hz. Each strip was stretched at a constant tensile speed of 10 mm/min until the failure occurred [23, 24]. Tensile test was performed within 6 hours of tissue harvesting to preserve mechanical properties [26]. During tensile test, strips were kept moist by dripping phosphate-buffered saline onto the surface of the tissue [27].

#### 2.6.3. Mechanical Data Analysis

The previous research has shown that when deformations of the materials were high enough, the engineering definition of strain and stress was no longer applicable and other definitions, such as true strain and true stress, should be used [28, 29]. Here, the rabbit ICAs were considered incompressible, meaning that the Poisson ratio is 0.495(*ν*) which enables us to report the true stress and strain values. This can be a great asset in obtaining the spontaneous dimensions of the tissue [23]. To calculate the true stress and true strain, we need to define engineering stress and strain, which depend on the initial dimensions of the tissue. The engineering stress is

(1) $$\begin{array}{c} \sigma_{E}=\frac{F}{A_{0}}, \end{array}$$

where *F* is the load and *A*_0_ is the initial cross-sectional area. The engineering strain is

(2) $$\begin{array}{c} \varepsilon_{E}=\frac{\Delta L}{L_{0}}, \end{array}$$

where Δ*L* is the change in length and *L*_0_ the initial length. Then, we define the true stress as

(3) $$\begin{array}{c} \sigma_{T}=\frac{F}{A}, \end{array}$$

where *A* is the current cross-sectional area. The true strain is defined by

(4) $$\begin{array}{c} d\varepsilon_{T}=\frac{dL}{L}, \end{array}$$

where *dL* is the instantaneous stretch and *L* the current length of the specimen. The assumption of the arterial incompressibility implies a zero change of volume during the tensile testing: *A* × *L* = *A*_0_ × *L*_0_; then,

(5) σTFA=F×LA0×L0=σE×L0+ΔLL0=σE1+εE.

The true strain is defined as the sum of all the current engineering strains; then,

(6) εT∫dε=∫L0LdLL=ln⁡LL0=ln⁡L0+ΔLL0=ln⁡1+εE.

**Figure 3:**
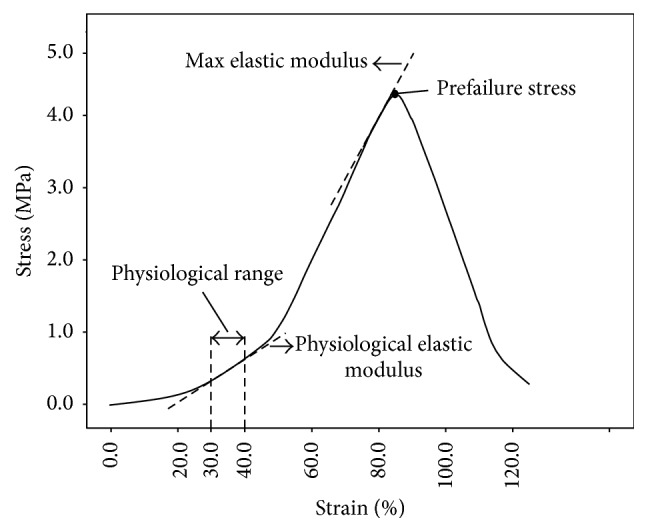
Principles of calculation of the physiological and maximum elastic modulus.

Furthermore, physiological elastic modulus (PEM) (in MPa), max elastic modulus (MEM) (in MPa), max strain (in %), and max stress (in MPa) were measured before the failure occurred. The max stress and max strain were defined as the maximum stress and strain prior to failure, respectively. PEM was defined as the slope of a straight line drawn between 30% and 40% strain [28, 29]. MEM is expressed as the slope of the linear steepest part of the load-strain curves up to the point of failure (Figure 3) [28, 29].

### 2.7. Histological Analysis

When finishing the whole process of intervention of CRM, one rabbit in each group was randomly chosen to be euthanized through air embolism. Then, the left ICA was harvested absolutely, washing with phosphate-buffered saline (PBS) buffer to remove the excessive fat and connective tissue. After embedding in paraffin, 5 *μ*m sections were prepared using a rotary microtome (Nikon Eclipse CI). And Hematoxylin and Eosin (H&E) was used to stain the tissue sections, observing typical atherosclerosis histology.

### 2.8. Statistical Analysis

SPSS version 20.0 was used for statistical analysis. All data are presented as the mean and standard error (SE). In order to determine whether the data was normally distributed, the Kolmogorov-Smirnov and Shapiro-Wilk tests were conducted. A two-way factorial ANOVA was used to determine the effects of CRM and modeling, and Fisher's PLSD [30] was then used for assessing differences in internal carotid tensile mechanical properties between the four groups. Before and after CRM/Non-CRM, the differences in internal carotid stenosis rate between the CRM-Model group and Non-CRM-Model group were compared with independent-samples* t*-test, respectively. The differences of internal carotid stenosis rate before and after CRM/Non-CRM were compared in the CRM-Model group and Non-CRM-Model group with Paired-Samples* t-*test, respectively. A value of *P* < 0.05 was considered significant.

## 3. Results

### 3.1. The Basic Situation of Rabbits

**Figure 4:**
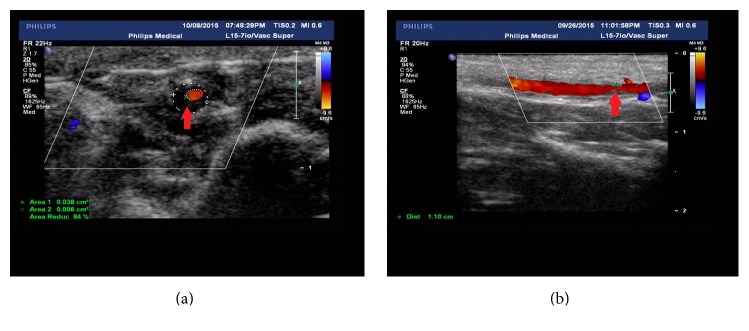
Color Doppler ultrasound demonstrating the atherosclerotic plaque in the ICA. The red arrows in (a) and (b) point to the places where the internal carotid atherosclerotic plaques are located.

In the process of modeling, 2 rabbits of model group (*n* = 24) died during the modeling surgery, and it was suspected that their deaths were related to anesthesia. One week after modeling surgery, another 3 rabbits of model group had diarrhea symptoms and finally died too. Subsequently, in 12th week after modeling surgery, the ICAs of the remaining rabbits (*n* = 19) in the model group all formed the atherosclerotic plaque (Figure 4). However, three of the remaining model rabbits' left ICAs were completely blocked by atherosclerotic plaques (Stenosis rate = 100%), so they were excluded. Therefore, there were 16 rabbits in the model group finally, and they were randomly divided into CRM-Model group (*n* = 8) and Non-CRM-Model group (*n* = 8). Before and after CRM/Non-CRM, there was no significant difference in all internal carotid stenosis rates between CRM-Model group and Non-CRM-Model group (Table 1). All of the normal rabbits in CRM-Normal group (*n* = 8) and Non-CRM-Normal group (*n* = 8) survived the process of the whole experiment.

### 3.2. Tensile Mechanical Properties

#### 3.2.1. Physiological Elastic Modulus (PEM)

**Figure 5:**
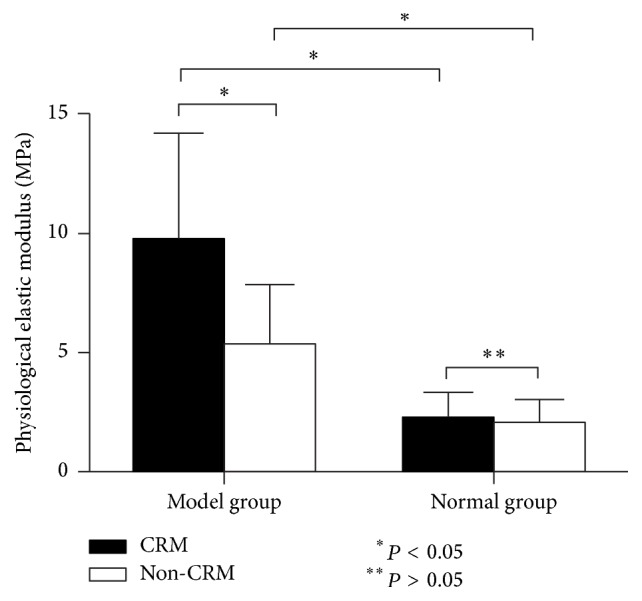
Physiological elastic modulus (MPa) of ICAs in the four groups (mean, SE).

As shown in Table 2, about PEM, the two-way ANOVA yielded a significant main effect of modeling (*P* = 0.00005) and a significant main effect of CRM (*P* = 0.025). What is more, the interaction between modeling and CRM was also significant (*P* = 0.038). Additionally, Fisher's PLSD test found that the values of PEM in the two model groups and in those rabbits treated with CRM were significantly higher than those treated without CRM (*P* = 0.003). But between the two normal groups, there was no significant difference (*P* = 0.896). Furthermore, the values of PEM in the CRM-Model group and Non-CRM-Model group were significantly higher than those in CRM-Normal group and Non-CRM-Model group, respectively (*P* = 0.000 and *P* = 0.021) (Figure 5).

#### 3.2.2. Max Elastic Modulus (MEM)

**Figure 6:**
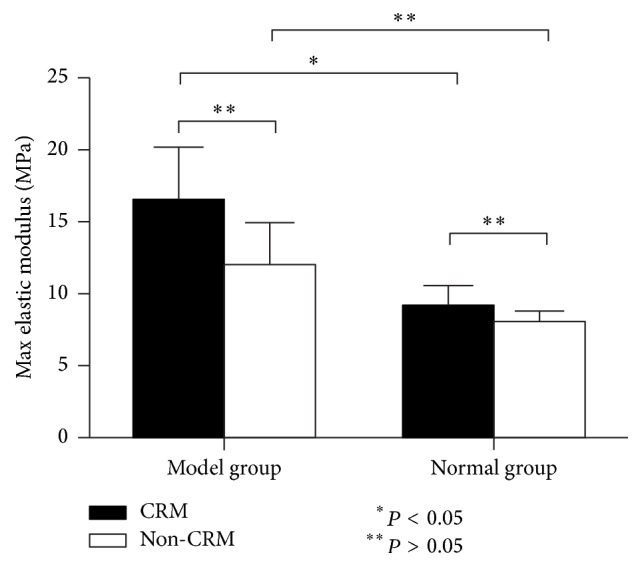
Max elastic modulus (MPa) of ICAs in the four groups (mean, SE).

As shown in Table 2, about MEM, the two-way ANOVA yielded a significant main effect of modeling (*P* = 0.028), while both of the main effect of CRM and the interaction between modeling and CRM were not significant (*P* = 0.265 and *P* = 0.501, resp.). Additionally, Fisher's PLSD test found that the value of MEM in the CRM-Model group was significantly higher than that in CRM-Normal group (*P* = 0.043) (Figure 6).

#### 3.2.3. Max Strain

**Figure 7:**
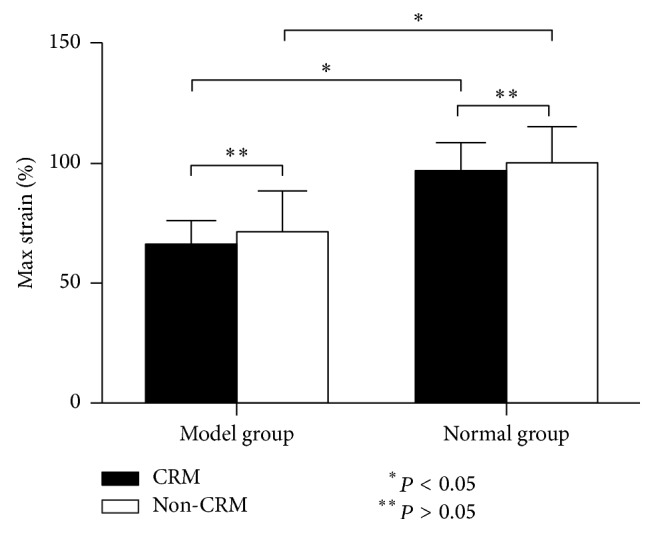
Max strain (%) of ICAs in the four groups (mean, SE).

As shown in Table 2, about max strain, the two-way ANOVA yielded a significant main effect of modeling (*P* = 0.00001). However, no significant main effect of CRM and modeling × CRM interaction was noted (*P* = 0.332 and *P* = 0.816, resp.). In addition, Fisher's PLSD test found that, whatever treated with CRM or not, the value of max strain in the two model groups was significantly lower than that in the two normal groups, respectively (*P* = 0.000 and *P* = 0.000, resp.) (Figure 7).

#### 3.2.4. Max Stress

**Figure 8:**
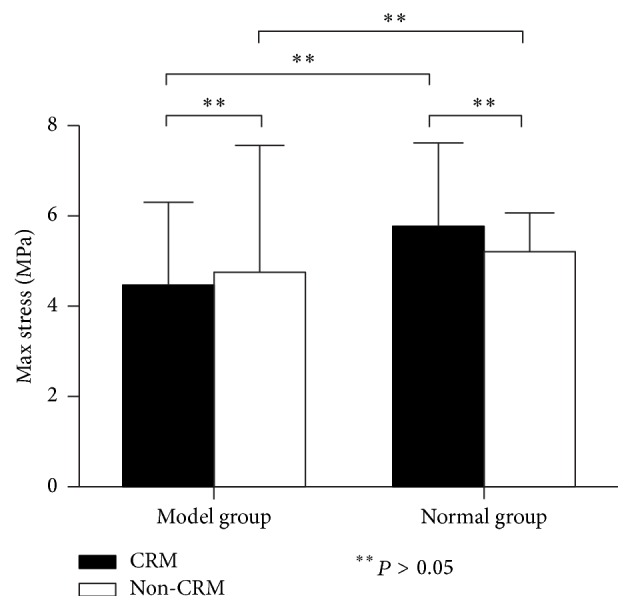
Max stress (MPa) of ICAs in the four groups (mean, SE).

As shown in Table 2, about max stress, the two-way ANOVA found that the main effect of CRM, the main effect of modeling, and the interaction between modeling and CRM were all not significant (*P* = 0.215, *P* = 0.863 and *P* = 0.520, resp.). Additionally, Fisher's PLSD test found that there was no significant difference in the value of max stress between the four groups (Figure 8).

### 3.3. Histological Observation

**Figure 9:**
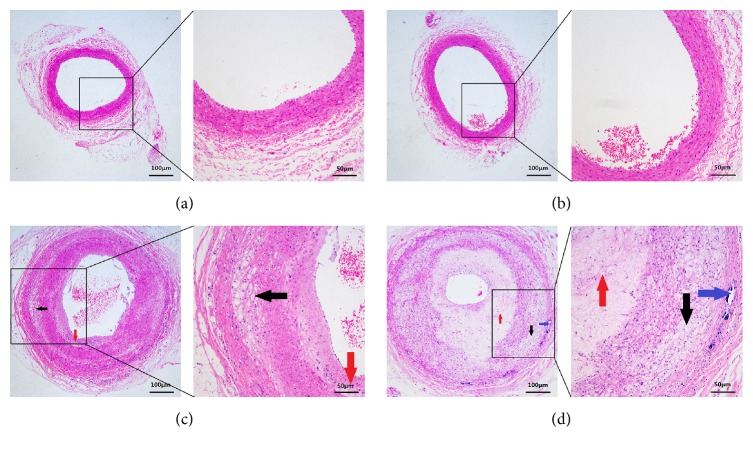
Morphological characteristics of the ICAs in the four groups. (a) and (b) Normal ICA with uniform thickness, regular fiber arrangement, and complete structure of tunica adventitia, media, and intima in the Non-CRM-Normal group and CRM-Normal group; (c) atherosclerotic ICA with slightly thickened intima (red arrows), edematous cells, thinly stained cytoplasm, and disordered cells arrangement (black arrows) in the Non-CRM-Model group; (d) atherosclerotic ICA with severe thickened and atherosclerotic intima, disordered fiber arrangement, and infiltration of foam cell (black arrows) and the calcified lesion at the junction between the tunica adventitia and tunica media (blue arrows) in the CRM-Model group.

The results of H&E staining of the ICAs in the four groups were shown in Figure 9. In the Non-CRM-Normal group and the CRM-Normal group, the ICAs' walls were normal with uniform thickness, regular fiber arrangement, and complete structure of tunica adventitia, media, and intima. The intima was a monolayer endothelial cell, and the overall structure of the artery was normal (Figures 9(a) and 9(b)). In the Non-CRM-Model group, the intima had a slight increase in the thickness, as shown by red arrows in Figure 9(c). Additionally, in the tunica media area, some edematous cells, thinly stained cytoplasm, disordered cells arrangement, and unclear overall structure appeared, as shown by black arrows in Figure 9(c). In the CRM-Model group, as shown by black arrows in Figure 9(d), there were severe thickening and atherosclerosis in the tunica intima of ICA. Moreover, atherosclerotic degeneration in the tunica media was observed, such as disordered fiber arrangement and infiltration of foam cell (black arrow in Figure 9(d)). What is more, the calcified lesion can be seen at the junction between the tunica adventitia and tunica media (blue arrow in Figure 9(d)).

## 4. Discussion

The results of this study provide some evidence that CRM may decrease the uniaxial tensile properties of rabbit arteriosclerotic ICA, but with no effect on normal group.

CRM is commonly used to treat neck pain in clinical practice [31–33]. Nevertheless, can CRM lead to the changes in the tensile properties of the ICA? There were few previous studies to explore the above question. This fundamental research has certain significance on clinical guideline, because it can investigate the changes in biomechanical property of ICA before and after CRM from the perspective of internal carotid tensile mechanical properties, which may provide an explanation of cardiovascular adverse events after the cervical spine manipulation in the clinical reports previously.

The biomechanical testing which is called uniaxial tensile test has been used in numerous experiments [22, 30, 34–38]. In previous reports, uniaxial tensile test of biologic tissue has been conducted for artery [34, 35], Achilles tendon [36], and even the carotid atherosclerotic plaque [37] samples. However, to our knowledge, few studies have assessed mechanical properties of ICA which had atherosclerotic plaque and had been treated with CRM. In spite of this, there were still many studies [14, 34] related to vascular biomechanic properties in previous studies which may help to explain the results of our experiments.

According to the analysis of the present study (Table 1, Figures 4–7), we found that both CRM and modeling were indeed the factors that affect the biomechanical parameters of the ICA. For the values of PEM, we found that, in the two model groups, the mean PEM of ICAs of those rabbits treated with CRM was 1.81 times as much as those treated without CRM. But for the healthy ICA, whether treated with CRM or not, the mean PEM was not statistically different. Karimi et al. [39] had found that the PEM and MEM of healthy human coronary arteries were 2.53 and 2.91 times higher than those of atherosclerotic coronary arteries, respectively. But the result of our study was quite different from theirs. Our result showed that, for the mean PEM of ICAs of those rabbits treated with CRM, atherosclerotic ICAs were 4.34 times as much as healthy ICA, and for those rabbits treated without CRM, atherosclerotic ICAs were 2.60 times as much as healthy ICA. Moreover, with regard to the mean MEM of ICAs of those rabbits treated with CRM, atherosclerotic ICAs were 1.80 times as much as healthy ICA. Elastic modulus is a datum that describes how a material will deform or break when subjected to a force or stress and is also a measure of the stiffness of arterial tissue. Therefore, in the present study, we can speculate that CRM might increase the stiffness of atherosclerotic ICA, but it did not affect the stiffness of healthy ICA. Previous research has shown that the increase in stiffness of arteries represents an early risk factor for cardiovascular diseases [14, 40, 41]; arterial stiffness has also been shown to be an independent risk factor for cardiovascular events [42]. So the increase of ICA's stiffness may be one of the reasons why CRM may lead to cardiovascular events.

Beside, the results of present study also showed that the max strain of ICA was mainly affected by internal carotid atherosclerotic plaque, but it was not affected by CRM. Max strain is a measure of extensibility of the tissue. Previous studies by Teng et al. [34] have shown that the mean stretch ratio at failure for all 72 human carotid specimens was 1.50 ± 0.22. Wand et al. [14] demonstrated that the normal vertebral artery could be stretched to 139% to 162% of their resting length before mechanical failure occurred. Karimi et al. [39] previously found that the max strain of atherosclerotic arteries were 34.61% lower than healthy ones, and similar results were obtained in our study. Our result revealed that, for the rabbits of those which have not been treated with CRM, the max strain of atherosclerotic arteries was 28.71% lower than healthy ones. And for the rabbits of those which have been treated with CRM, the max strain of atherosclerotic arteries was 30.98% lower than healthy ones.

Recently, it is generally recognized that the mechanical properties of blood vessels are closely related to the constituent and structure of the vessel wall [43]. The typical pathological changes associated with the development of atherosclerotic plaques within arterial vessels will result in significant alterations to the mechanical properties of the diseased arterial wall [44–46]. We speculate that CRM may aggravate the severity of internal carotid atherosclerosis and further affect the tensile mechanical properties of ICA. And the pathologic results of the present study were consistent with the hypothesis. As shown in Figure 9, it can be obviously found that the internal carotid atherosclerosis in CRM-Model group was much more severe than that in Non-CRM-Model group. These pathological findings may explain why the tensile properties of ICA in CRM-Model group were lower than those in the Non-CRM-Model group and normal groups.

The present study also has certain limitations. First, although CRM were carried out by the same osteopath who had many years of CRM experience, due to the limitations of the experimental conditions and the feasibility of the operation, there is no specific quantification of the operation, such as quantifying the rotation strength and velocity in the operation process; second, several specimens broke at or near one of the clamps in the process of tensile test, most likely due to stress concentrations. In these cases, it is likely that the max stress was underestimated; third, the circumference of the transverse section of rabbit ICA was very short, it was too hard to do the uniaxial tests in circumferential direction, so we just did the uniaxial tests in longitudinal direction.

## 5. Conclusions and Directions for the Future Study

In conclusion, we can draw the following conclusions: (1) CRM may decrease the tensile mechanical properties of rabbit arteriosclerotic ICA and this effect may lead to rabbits developing adverse complications; (2) CRM has no significant effect on the tensile mechanical properties of the ICA in normal rabbits; (3) for patients with carotid atherosclerosis, for safety reasons, it is best to avoid to do the CRM in these patients.

However, why does CRM have an influence on the biomechanical properties of atherosclerotic ICA? We put forward a hypothesis that CRM may change haemodynamics of ICA (such as wall shear stress), and once wall shear stress in ICA changed, the force of blood flowing to ICA endothelium will change. Due to the presence of atherosclerotic lesions of the ICA, the wall shear stress is more likely to change organizational structure of vascular endothelia and then change the biomechanical properties of the vessel. To our knowledge, previous studies have been conducted to demonstrate the effects of changes in vascular haemodynamics on vascular endothelial tissue architecture and the relationship between the changes of vascular endothelial tissue architecture and the biomechanical properties of blood vessels [45–51]. Den Dekker et al. [47] presented a novel method to induce atherosclerosis in New Zealand white rabbits using a flow altering device, and they found that both low and oscillatory shear stresses were capable of inducing atherosclerosis in a large animal model. Cheng et al. [50] also suggested that atherosclerotic lesion size and vulnerability were determined by patterns of fluid shear stress. Mulvihill et al. [45] demonstrated that the plaques with a higher calcification than lipid content produced a stiffer mechanical response than those with higher lipid content, which conversely displayed a softer response.

Therefore, on the basis of previous studies and the present study, there are still two researches which are very worthy of further study. And these two studies have been ongoing by us. One is to demonstrate the effect of CRM on the haemodynamics of ICA (especially atherosclerotic ICA). The other is to study the effect of CRM on the stability of carotid atherosclerotic plaque. We believe that we will provide a better explanation for the pathogenesis of cardiovascular disease after CRM through the present study and our ongoing researches.

## Figures and Tables

**Table 1:** Internal carotid stenosis rates of model groups before and after CRM/Non-CRM.

Group	*n*	Internal carotid stenosis rates (%)			*P*
		Before CRM/Non-CRM	After CRM/Non-CRM	*D*-value	
CRM-Model group	8	83.88 ± 7.10	81.50 ± 12.76	−2.375 ± 10.43	0.540
Non-CRM-Model group	8	75.38 ± 14.44	89.75 ± 12.38	14.375 ± 17.60	0.054
*P*		0.157	0.211		

**Table 2:** Biomechanical parameters of rabbits' ICAs in the four groups.

Trait	CRM-Model group	Non-CRM-Model group	CRM-Normal group	Non-CRM-Normal group
Physiological elastic modulus (MPa)^abc^	9.68 ± 4.55^df^	5.34 ± 2.51^g^	2.23 ± 1.11	2.05 ± 1.02
Max elastic modulus (MPa)^b^	16.51 ± 10.38^f^	12.05 ± 8.06	9.16 ± 4.01	8.04 ± 1.97
Max strain (%)^b^	65.54 ± 10.15^f^	71.43 ± 16.53^g^	96.52 ± 12.07	100.14 ± 20.09
Max stress (MPa)	4.43 ± 1.87	4.76 ± 2.81	5.77 ± 1.84	5.19 ± 1.94

Two-way ANOVA results:

a denotes significant *P* < 0.05 CRM × Model interaction.

b denotes *P* < 0.05 significance of model versus normal controls (model effects).

c denotes *P* < 0.05 significance of CRM versus Non-CRM (treatment effects).

d denotes *P* < 0.05 significance of comparisons for CRM versus Non-CRM within model group.

f denotes *P* < 0.05 significance of comparisons of model group versus normal group treated with CRM.

g denotes *P* < 0.05 significance of comparisons of model group versus normal group treated without CRM.
